# Access to Water, Sanitation and Hygiene Services and Other Preventive Measures against COVID-19 among People with Disabilities, Dodoma, Tanzania

**DOI:** 10.4269/ajtmh.21-0756

**Published:** 2022-07-25

**Authors:** Hussein Mohamed, Elizabeth Wamera, Wilhelmina Malima

**Affiliations:** ^1^ Department of Environmental and Occupational Health, Muhimbili University of Health and Allied Sciences, Dar es Salaam, Tanzania;; ^2^ 1eme etage, Aram Siga, Almadies, Dakar, Senegal;; ^3^ Sanitation and Water Action (SAWA), Tanzania, Opportunity Tanzania Building, Dar es Salaam, Tanzania

## Abstract

Access to Water, Sanitation and Hygiene (WASH) among people with disabilities is a great concern in developing countries. During COVID-19 pandemic in 2020, in addition to other preventive measures the government of Tanzania invested heavily on handwashing facilities in public places. However, the interventions were mostly for the general population. A big question was the appropriateness of the preventive measures to address different kinds of disabilities. The study was conducted to assess access to WASH and other preventive measures against COVID-19 among people with disabilities. This was a qualitative study where a total of 16 key informant interviews and nine focus group discussions were conducted. The study was carried out in seven districts of Dodoma region, Tanzania. Findings show that there were inadequate WASH and other COVID-19 preventive measures designed specifically for people with disabilities against the pandemic. Many people with disabilities experienced challenges in accessing adequate water for handwashing and using handwashing facilities installed for general population. Also they received inadequate health education and timely communication on COVID-19 preventive measures in addition to challenges in keeping distance and accessing and use of face masks and sanitizers. People with mobility, hearing, and vision impairments were mostly affected. There was no representation of people with disability at the national COVID-19 task force. Specific programs for people with disabilities to address access to WASH and other preventive measures against COVID-19 would address most of the identified challenges.

## INTRODUCTION

People across the world are currently facing an unprecedented global health emergency due to the outbreak of COVID-19. COVID-19 is caused by a virus (SARS-CoV-2) and is associated with acute respiratory syndrome. All ages are affected but observations so far indicate that older persons and those with compromised immunity are at a higher risk of acquiring infection.^1^ Tanzania, like many other countries, has reported cases of COVID-19. The last time for Tanzania to report cases of COVID-19 was in May 2020, where there were a total of 509 confirmed cases with 21 deaths.^1^

The Government in collaboration with Development Partners, Non-Governmental Organization, and private institutions worked tirelessly to make sure that the disease transmission is prevented and all confirmed cases were treated accordingly. Some of the preventive measures include screening all arrivals and departures at points of entry and instituting appropriate actions. Other measures include provision of health education on hand hygiene, keeping physical distance, and use of face masks. Access to safe Water, Sanitation and Hygiene (WASH) is necessary for protecting human health during all infectious disease outbreaks including COVID-19. WASH interventions especially frequent and correct hand hygiene is one of the most important measures to prevent infection with the COVID-19.^1^ This calls for WASH practitioners to work toward enabling all people including people with disabilities to access water and other sanitation facilities so that they can practice frequent and regular hand hygiene necessary for the prevention of COVID-19. While accessibility to Water, Sanitation and Hygiene services is critical to the prevention of COVID-19 and other communicable diseases, people with disabilities may be experiencing challenges in access and use of WASH facilities. People with disabilities face substantial challenges to meeting their WASH needs, particularly in using services autonomously, consistently, hygienically, with dignity and privacy, and without pain or fear of abuse.^2^ Many interventions are planned for a general population, only few focuses to people with disabilities.

The COVID-19 pandemic has disrupted many aspects of lives; its impacts are more intense especially for people with disabilities. According to the Laws of United Republic of Tanzania (URT), the Persons with disabilities Act, 2010, defines a person with disability as a person with a physical, intellectual, sensory, or mental impairment and whose functional capacity is limited by encountering attitudinal, environmental, and institutional barriers.^3^ It is estimated that there are about 4.5 million people with disabilities in Tanzania. In 2014, the Tanzania National Bureau of Statistics (2014) reported that there were a total of 2,225,671 people with disabilities aged between 7 years and above.^4^ The groups of disabilities are categorized into albinism, seeing, hearing, walking, remembering, self-care, and communicating.^4^ People with disabilities may experience different difficulties in accessing WASH and other services depending on the nature of disability.

All the general challenges that come with the pandemic certainly apply, but there are additional barriers. Getting information can be more difficult for people with vision, hearing, and even cognitive disabilities as popular news sources may not be accessible, especially when information is changing quickly. Adopting recommended public health strategies such as social distancing and washing hands is also a challenge. For example, frequent handwashing is not always feasible for people with certain types of physical disabilities. Those who have personal aides and caregivers also need to be considered, as they cannot participate in social distancing in the same way that others are. Moreover, equitable access to healthcare is a long-standing barrier worsened by COVID-19. For example, the use of personal protective equipment including masks can make communication more difficult for people with hearing impairment because some of them read lips as a form of communication.

Worldwide, the research on the challenges people with disabilities face regarding access to WASH and other health services is adequate. Despite the fact Tanzania has enacted an Act “The Person with disabilities Act, 2010”, and also has ratified the United Nations Convention on the Rights of Persons with Disabilities, much has not being put into action. For example, a study by Mesiäislehto et al. in accessing sexual and reproductive health services at the intersection of disability and female adolescence in Tanzania revealed that adolescent females with disabilities experience challenges in terms of approachability, acceptability, availability, affordability, and appropriateness.^5^ It is important therefore, to assess challenges facing people with disabilities with regard to COVID-19 prevention so as to inform the public health response. The study was designed with main objective to assess the access to WASH services among people with disabilities, identify WASH-related measures and innovations against COVID-19, document existing challenges and systemic gaps.

## MATERIALS AND METHODS

### Study design.

This was explorative cross-sectional qualitative study that was conducted to assess access to water, sanitation and hygiene services and other preventive measures against COVID-19 and the associated challenges among people with disabilities.

### Study area.

The study was conducted in seven councils of Dodoma Region namely Dodoma Municipal Council, Chamwino District Council (DC), Bahi DC, Kongwa DC, Kondoa Town Council (TC), Mpwapwa DC, and Chemba DC. Dodoma region was chosen because it is among the regions with large number of people with disabilities.^3^ The assessment was done only at district headquarters.

### Study population and sampling.

The main study population of interest was people with disabilities in each district. The disability in this study was defined as vision impairment, deaf or hard of hearing, and physical disability. Sampling was done conveniently where the social welfare officers informed participants to come to a particular place to participate in the study. Each focus group discussion (FGD) comprised of 8–10 participants both male and female with mixed kinds of disabilities. The FGDs were carried out in public places mainly DC offices or schools.

### Data collection and analysis.

The study used a qualitative method of data collection. Data collection was carried out by qualified and experienced facilitators where key informant interviews (KIIs) and FGDs were conducted. A total of 16 KIIs were carried out at two levels, national and at the subnational level—the Council level. At national level, KIIs were conducted with focal person on social welfare issues at the President’s Office, Regional Administration and Local Government (PORALG); and the Director dealing with Labor, Youth, Employment and Persons with Disabilities at the Prime Minister’s Office. At the council level, the KIIs were conducted with environmental health officers and social welfare officers whose roles include health education and promotion in council.

A total of nine FGDs were carried out, one in each district with a group of people with disabilities and the other two with leaders representing associations of people with disabilities, one in Dodoma and the other in Dar es Salaam. All associations of people with disabilities in Tanzania are under one umbrella called Tanzania Federation of Disabled People’s Organizations –SHIVYAWATA (in Kiswahili acronym). These associations include Tanzania Albino Society (TAS), Tanzania League of the Blind (TLB), Tanzania Association of the Physically Handicap (CHAWATA), Tanzania Association of the Deaf (CHAVITA), Tanzania Association of the Deaf—Blind (TASODEB), and Tanzania Users and Survivors of Psychiatric Organization (TUSPO).

All KIIs and FGDs sessions were audio recorded with the consent of participants for translation and subsequent transcriptions. Kiswahili, a national language, was used during KIIs and FGDs. Respondents were provided with a code to ensure anonymity in the notes. Following discussions, the facilitator and note taker finalized the notes daily. All audio recordings were then transcribed verbatim and translated from Kiswahili to English.

### Data analysis.

Content analysis was used to analyze the data. Qualitative content analysis is the approach that allows for the subjective interpretation of the content of text through a systematic process of coding and identifying themes or patterns. The method is flexible and pragmatic for developing and extending knowledge of the human experience of health and illness. For all the interviews from FGDs and KIIs data were digitally recorded and afterwards transcribed verbatim. Transcriptions were entered into the program Open Code to support the coding process.

### Ethical clearance and consent.

Ethical clearance was sought from Research and Publications Committee of Muhimbili University of Health and Allied Sciences (MUHAS). Permission to conduct the study was obtained from President’s Office-Regional Administration and Local Government (PORALG) who wrote a letter to District Executive Councils to introduce the research team. Both oral and written consent were sought from participants who were eligible. Only those who consented to participate were recruited in the study.

## RESULTS

### Demographic characteristics of respondents.

Data from seven DCs of Dodoma region indicate that there are a total of 1,490 people with disabilities out of 2,216,813 population equivalent to 0.67% (Table 1). Kondoa TC had more people with disabilities (*N* = 747) with a proportion of 1% followed by Bahi DC (*N* = 2574) with a proportion of 0.95%.

**Table 1:** Proportion of people with disabilities by district council

District council	Total population	Total number of people with disabilities	Proportion of people with disabilities
Dodoma City Council	490,000	1,230	0.25
Chamwino	418,704	2,110	0.50
Bahi	271,500	2,574	0.95
Chemba	308,685	2,409	0.78
Kondoa TC	74,718	747	1.00
Mpwapwa	312,000	2,756	0.88
Kongwa	341,206	3,083	0.90
**Total**	**2,216,813**	**14,909**	**0.67**

TC = town council.

A total of 102 people with disabilities participated in this study (Table 2). Their ages ranged from 24 to 77 years. Females were 46 while the remaining were males. Majority of participants had physical disability (*N* = 34) followed by albinism (*N* = 27) whereas those with hearing impairment were 22. Other kinds of disabilities and the number of participants are summarized in Table 2.

**Table 2:** Description of the study participants

Age group (years)	Total
21–30	23
31–40	41
41–50	23
51–60	9
61–70	4
71–80	2
Total	102
Kind of disability	Total
Hearing impairment	22
Albinism	27
Visual impairment	11
Partial visual impairment	5
Albinism plus partial visual impairment	3
Physical disability	34
Total	102

A review of reports indicates that there were more than 15,000 people with disabilities in all seven councils of Dodoma region. Kongwa and Bahi DCs had more population with disabilities than other councils (3,083 and 2,574 respectively).

### Availability, accessibility, and use of WASH services.

Regarding availability and accessibility on WASH services during COVID-19, findings show that people with disabilities experienced challenges in accessing clean and safe water for handwashing. It should be noted that although people with disabilities might have had previous challenge of water supply, it was during COVID-19 that the demand for water increased especially after a continuous education on handwashing with running water and soap, one of the primary measures to prevent COVID-19 infections. One respondent during FGD had the following to say: “*Water sources are located very far and, in that case, I cannot walk long distances to fetch water for my needs, I have become dependent to my neighbors or when my children visit me”* (a man with physical disability, FGD Bahi DC). Buying water from vendors is used as a coping mechanism to those with water supply challenges. However, only few can afford to buy even a single bucket of water every day. And if someone is able to buy, the water will be used mainly for cooking and drinking, not for handwashing. One respondent had this to say “*I do not have any income, so I couldn’t afford to buy water every day since one bucket of 20l costs up to 500 Tanzania Shillings (equivalent to USD 0.22) and so it was very hard for me*.” (a man with physical disability, FGD, Mpwapwa DC). In addition to difficulties experienced by people with disabilities to access water for hand hygiene, it was further revealed that practicing hand hygiene was also a challenge especially in public places. When COVID-19 was reported in the country, the government in collaboration with partners and the private sector invested heavily on handwashing infrastructure in public places such as at the markets, bus stands, training institutions, hospitals, and schools to make sure that people can access hand wash facility wherever they are. Hand wash facilities ranged from simple bucket or large plastic containers fixed with a tap to more complex permanent structures with several taps fixed on the walls. It was reported that most of those hand wash facilities were not user friendly to most people with physical disabilities. One participant explained that: *“At times I wanted to wash my hands but handwashing facilities were not suitable for my physical disability so I had to rely on sanitizers”* (a man with physical disability, FGD, Mpwapwa DC). Similarly, another FGD participant narrated that *“One day I went to the bus station and I wanted to wash my hands, I discovered that the handwashing facility was above my reach and so I was not able to use it and therefore I could not prevent myself from COVID-19 through handwashing” *(FGD, a man with physical disability, FGD Dodoma MC). Generally, people with disability reported to experience challenges in using hand wash facilities despite their availability in many public places. The main reason was that many of the installed hand wash facilities were not friendly to them. Our observations indicate that some hand wash facilities were installed at 2 m above the ground that was beyond the reach of a person with mobility impairment, for example.

Participants reported to use other means of hand hygiene for prevention of COVID-19 mostly use of sanitizers. It was reported that sometimes people with disabilities were provided with free sanitizers by the local government and by Non-governmental Organizations (NGOs). Some of the people with disabilities were unable to use them. It was noted that free sanitizers were not provided routinely and did not reach every person with disability. Those who were able to use sanitizers had to buy at local shops at a minimum price of about Tanzania shillings 2,500 (USD = 1.086). This price was reported to be on a high side that even those who wanted to use sanitizers they could not afford when needed. One responded had this to say *“sanitizers were very costly and sometimes I couldn’t manage to buy them*” FGD, (a man with physical disability, FGD Dodoma MC). There was one other limitation of using sanitizers among people with disabilities. It was reported that many people with disabilities, especially physical disabilities did not see the importance of using sanitizers as one of them was quoted saying *“Even if you apply sanitizer you still have to crawl on the ground and touch surfaces that might be contaminated by Corona virus, so it is like doing nothing”* (a man; with physical disability, SHIVYAWATA).

Regarding access to sanitation facilities it was reported that people with disability did not feel safe using public toilets in public places because there was a possibility of getting infected with COVID-19 through touching door knobs and other surfaces around the toilet. One of the respond narrated that “*when I am in the market, I am so scared to go to toilet because everyone touches the door, I do not know if all practice hand hygiene, I might get COVID-19”* (FGD, a woman with disability, Dodoma MC).

### Health education services on preventive measures against COVID-19.

Adherence to COVID-19 preventive measures depends on getting right information at the right time. Adherence to COVID-19 preventive measures among people with disabilities was hindered by inadequate health education and timely communication. At first, health education and promotion program was designed for a general population without taking into consideration different disabilities within the community. As such, many people with disabilities were not reached with right information timely on what to do to prevent themselves from COVID-19 infections. The main channels of communication used to educate general public include local radios, magazine, poster and leaflets, and public announcements. Some people with disability were not reached with the information depending on the kind of disability and the channel of communication used. One respondent had this to say “*For example a person like me who is albino have poor sight, so when these posters and leaflets are of small sized letters, it makes it hard for me to read”*, (a woman with albinism, FDG Chemba). This was caused by lack of specific program to address COVID-19 and people with disabilities from the beginning of the pandemic. However, it was revealed that many people with disabilities relied on family members to inform them on what was happening regarding COVID-19 and preventive measures. “*I get challenges in understanding what is being communicated about COVID-19, thank to my boy who informs me many issues*” (A woman with hearing problem, Dodoma Council) narrated. Providing health education to people with disabilities was reported to be a challenge taking into account different types of disabilities such as physical, visual, and hearing impairment. One of the responded said that *“there is no designated specific information about COVID-19 outbreak that is meant for us; we have different types of disabilities such as physical, visual and hearing impairment; and so everyone needs a special attention when it comes to information sharing, and so the government should consider us when designing messages on COVID-19*” (Man with hearing problem, FGD Kondoa TC). It was therefore, concluded that at the beginning of pandemic, there was no health education program specifically designed for people with disabilities as one of the responded was quoted saying that “*We didn’t get any special information on the Corona outbreak that were meant for people with disabilities, however, we were provided leaflets with braille very late, it was almost the end of first wave of COVID-19 in our country”* (man, visually impaired, FGD Kongwa DC). Therefore, inadequate timely information on COVID-19 to people with disabilities affected adherence to preventive measures in many aspects.

### Other preventive measures against COVID-19.

Regarding availability and use of face masks during COVID-19 among people with disabilities, while some participants of FGD reported to use facial masks to prevent them from COVID-19, others said it was hard for them to get face masks since they did not have income to buy. Although it was reported that people with disabilities were provided free face mask by the government, they were not enough for daily need and thus they had to buy in local shops. It was reported that an average price for one medical mask was sold at Tanzania Shilling 500, equivalent to USD 0.22 and that many could not afford to buy always. One of them said: *“I could not buy face mask because I could not afford them. And sometimes I had very little amount of money, so I prioritized food over the face mask”,* (a woman with physical disability, FGD Dodoma City Council).

Keeping social distance was one of the preventive measures for COVID-19. People with disabilities, like others, were told to keep distance at least 1 m from one another. It was revealed that participants had mixed feelings about this preventive measure. Some of them reported to be able to stay at least 1 m apart from people (apart from family members) since most of the time they stayed at home. One of them said that “*I do not have much to do in town, so I was just staying home during all this time of COVID-19”* (a man, FGD, Dodoma CC). On the other hand, people with disabilities experienced difficulties to adhere to this measure. One of the respondents had this to say *“some of us need an assistant*, *how do you keep social distance while you are supposed to walk side by side by someone to help you*?” (FGD, a woman with physical disability, SHIVYAWATA).

As with regards to WASH innovation, the finds revealed that there was none specifically designed to enable people with disabilities practice hand wash except for a general population.

### Main kinds of disabilities that limit access to WASH services and information.

During FGD, we wanted to know the main kinds of disabilities that limit access to WASH and information on COVID-19. Majority of respondents mentioned blindness and physical disabilities (disabilities of hands and legs) as the main kinds of disabilities that limit them. One of the respondents had this to say *“Yes, I cannot wash hands due to my physical disability”* (a woman with physical disability, FGD Chamwino DC). Furthermore, people who have impaired hearing and seeing experienced more challenges in accessing information on COVID-19. What we observed during our study was a technological gap that means much as hand wash facilities were installed in many public places, they were not user friendly to many people with disabilities. Likewise, the communication channels to educate the public on COVID-19 preventive measures were not in favor to some people with certain kind of disabilities.

### Challenges reported by health education and promotion officers.

During the study, we also conducted KIIs with district Environmental Health officers and Social Welfare officers to explore on the challenges they face during provision of health education on COVID-19 preventive measures to people with disabilities. Majority of them reported to experience budget constrains when they wanted to reach people with disabilities. They reiterated for example that, it was sometimes costly to hire an interpreter who could assist to communicate to people who had hearing and talking disabilities. Likewise, it was regarded expensive to prepare *Braille letters* for people with vision disability when preparing information education and communication (IEC) materials like leaflets. Generally, it was informed that little budget was allocated for people with disability to address most of their needs. One respondent had this to say “*there is very little budget allocated for people with disabilities at district level, sometimes we do receive WASH funds from the Ministry of Health but they come with clear guidelines on how to use them,”* (KII, Environmental Health Officer, man, Bahi DC). One of the social welfare worker narrated that “*we try to reach them all, but sometimes it is difficult: it is not realistic to plan for a visit to remote village where there is only one person with disability: we can only do so when we have other program in that area”* (Woman, Social Welfare Officer; Kondoa TC, KII).

Another challenge reported by health education and promotion officers with regard to providing health education on COVID-19 to people with disabilities was lack of accurate statistics of people with disabilities. There was no realistic number of people with disabilities and the kind of disabilities in each district that we visited. It was difficult to plan any intervention without knowing the number of people with disabilities and the kind of disabilities, health promotion officers said. They further narrated that, *“we do not know the total number of people with disabilities, and this makes us unable to have a realistic intervention program”. (*KII, A Woman Health Officer, Chamwino).

### Challenges reported by SHIVYAWATA.

Challenges faced by people with disabilities in accessing WASH services and health education regarding COVID-19 preventive measures were supported by SHIVYAWATA leaders. They reported that some of the participants were able to use WASH services but most of them were not able to use and access them because the WASH facilities installed in many public places during COVID-19 were not friendly for each kind of disability. One of the leaders said that “*WASH facilities are installed everywhere in public places but not all people with disability benefit*” (FGD, man with physical disability, national SHIVYAWATA*).* The other main challenge was availability of water that was exacerbated by high demand of handwashing during COVID-19 pandemic. One SHIVYAWATA leader had this to say “*on behalf of my group CHAWATA I can assure you that access and availability of water among people with disabilities is critical problem during COVID-19*” (FGD, a woman with physical disability, SHIVYAWATA).

### Representation of people with disability in national COVID-19 response team.

As part of an effort to address COVID-19 pandemic, the government formed a national response task force with several subcommittees such as Infection Prevention and Control (IPC), WASH, Case Management, Risk Communication, and so on. This study explored on whether people with disabilities were represented and what were their roles and responsibilities in the national task team. Findings revealed that there was no representation of people with disabilities in the national task force. The main reason for not having a representative was reported to be lack of funds since it would need to pay one more person who would assist the person with disability all the time. One respondent was quoted saying that “*the financial constraint was the main limitation to accommodate a representative from people with disability who would need another person to assist him/her*” (KII, a man, Prime Minister’s Office, Department of works, youth, employment, and people with disabilities).

## DISCUSSION

The study has reported an important public health issue with regard to COVID-19 preventive measures among people with disabilities. People with disabilities are reported to face many challenges even during the times when there is no outbreak of the diseases. We have seen from this study that it was difficult for most of them to observe most of the preventive measures against COVID-19 infections. We did not find out how many were diseased and died due to COVID-19 since it was beyond the scope of this study.

Similar findings on challenges that people with disabilities face with regard to access WASH services have been documented in other studies. Most studies have indicated that people with disabilities experience a multitude of challenges in accessing different services. The Christian Blind Mission International Australia highlighted challenges faced by people with disabilities in accessing WASH services and include the fact that some WASH facilities are physically difficult to reach or use; some are inaccessible or inapplicable hygiene information; stigma or discrimination; have specific hygiene needs, particularly if they have difficulty with their movement and/or if they use their hands to move, navigate, or communicate; the need to touch things to obtain information from the environment.^4^ In one study, it was revealed that contributors to limited access to healthcare services among people with disabilities include access barrier to healthcare (65.6%), medical equipment barriers (78.14%), communication barriers (66.3%), and physical barriers to healthcare (55.5%). Among the group reported to have experienced physical barriers, half (50.8%) constituted persons with physical disabilities whereas 48.5% were visually impaired.^6^ Across sectional study to determine access to WASH among people with disabilities in four countries namely Bangladesh, Cameroon, India, and Malawi indicated that the main challenge is not access to WASH at household level, rather poorer quality of access within their households. In the same study it was reported that 23–80% were unable to collect water themselves whereas 53% reported to come in contact with faeces when accessing sanitation facilities.^2^

Challenges in accessing WASH services are associated with the type of disability of individuals. In many cases not all face the same set of barriers.^7^ In one study, it was reported that the main factors were physical impairment and greater disability severity; lower socioeconomic status and physical or self-care limitations.^8^ Similar findings are reported in our study. Regarding age, in one study in Guatemala it was reported that among people with disabilities, older adults were more likely to experience difficulties in hygiene and sanitation than younger people with disabilities.^9^ In addition, it was found that the number and intensity of the barriers faced by an individual to access WASH increases by being female, being from an urban area, and having limited wealth and education.^7^ This is an important piece of information although our study did not correlate demographic characteristic with intensity and severity of challenges people with disabilities face. Other factors that significantly affect access to adequate WASH include body function limitations such as incontinence, pain and inability to communicate WASH needs.^7^ Generally, people with disabilities are at increased risk of many infectious communicable diseases that needs special attention. Depending on the nature of the disabilities one has, interventions to address such challenges can be at family level, institutional level, or national level.

Access to correct information is a key to practice preventive measures on COVID-19. Studies indicate that people usually perform better when they are well informed.^10^ It is through correct and timely information that people can be able to take appropriate actions to protect themselves from things that are threat to their health and livelihood. Studies indicate that people with disabilities shows a disparity of health services compared with ordinary people. This is contributed by communication barriers and also a reduced involvement and participation in health promotion activities^11^^,^^12^ as compared with general population which are critical for prevention of COVID-19 and other communicable diseases. It is because of this that SHIVYAWAT asked for capacity strengthening so that people with disabilities could be able to educate themselves with the notion that “nothing for us without us.” A review study on barriers to access and use of health information by individuals with Intellectual and Developmental Disability (IDD) revealed six barriers to information access, which include communication skill, patient engagement, and satisfaction; training/education; attitude and knowledge of healthcare providers; being excluded from health promotion and research; and quality of accessing healthcare services.^13^

Communication barriers among people with disabilities are a major obstacle in getting healthcare services. Studies indicate that the most affected individuals are those with speech and hearing impairments.^6^^,^^14^^,^^15^ Same findings have been reported in our study that people with hearing problem and those with physical disabilities were more likely to be affected. A big communication barrier between healthcare workers and people with disabilities is that most of them neither understands nor appropriately communicates in sign language, yet, the sign language interpreters are not easily available.^7^^,^^15^ Evidence indicates that indirect communication is also a challenge for certain disabilities. For example, individuals with visual impairments cannot access information in pictures and on flip charts and leaflets or on any other written or print materials.^16^^,^^17^ This was among the main challenges that affected people with disabilities reported our study. Likewise, people with hearing impairments cannot hear or access messages delivered via radio, TV, or any other media that involves sound.^18^ As mentioned earlier, information is power; information makes people to make right choices, information makes people find a way to respond to diseases. Limited access to timely information is a hindering factor toward improved health among people with disabilities. It is due to this fact that people with disability could not be able to follow simple instruction messages such as keeping physical distance of at least 1 m from others, avoid crowds and close contact, wear a properly fitted mask when physical distancing is not possible and in poorly ventilated settings, clean your hands frequently with alcohol-based hand rub or soap and water, cover your mouth and nose with a bent elbow or tissue when you cough or sneeze, and dispose of used tissues immediately and clean hands regularly. Such educative messages were very important public health measures against COVID-19 pandemic.

Lack of representation of people with disabilities in the national task force for COVID-19 pandemic might have denied their contributions in planning for COVID-19 preventive measures and other services. As reported in this study, people with disabilities experienced many challenges during COVID-19 pandemic that we believe could have been addressed by their representation in the national task force. Interventions such as hand wash facilities which were among the top priorities might have considered physical limitations and other kinds of disabilities rather than having uniform interventions for a general population. The reason that was provided by officials as to why there was no representation of people with disability in the national task force would not outweigh the importance of having them in the team.

Local health officers and social welfare officers who were expected to provide support to people with disabilities with regard to COVID-19 preventive measures reported to experience challenges in reaching them caused mainly by lack of funds. It is a fact that people with disabilities live with their families in different areas, some far away from district headquarters making difficulties for district officials to reach them regularly. This can be solved by each district having a budget line dedicated to people with disabilities so that whenever health promotion officers want fuel for example, it is becomes easy for them to get and travel to different places within the district. In addition, since people with disabilities are part of the general population as they do not live in isolation, both community and family members have a big role to play to make people with disabilities live a healthy life.

## CONCLUSION

This study concludes that people with disabilities had difficulties in accessing WASH services as one of the critical preventive measures against COVID-19. The challenges experienced in accessing WASH and information services on COVID-19 varied with the kind of disabilities; those with impaired vision or blindness and those with physical disabilities were mostly affected. Most of the WASH interventions designed by the government during COVID-19 was for the general public, very little were designed for people with disabilities, yet, not all took into account all kinds of disabilities. People with disabilities received appropriate information on preventive measures against the pandemic much late compared with general population. Many were unable to get adequate information because channels of communication were not suitable to all kinds of disabilities.

The study recommends that access to COVID-19 preventive measures is a right to all people in the communities including people of disabilities. When developing intervention programs, consideration should be made to make sure that they are appropriate to people with disabilities, be consistently provided, and accessible all the time. With regard to communication, messages whether written or audio should be designed to suit all types of disabilities. Additionally, the government should create friendly environment for people with disabilities to do economic activities that will enable them generate income.

## STUDY STRENGTHS AND LIMITATIONS

- **Strength.**
  - Findings of this study can generalize challenges people with disabilities face in accessing other basic services.
- **Limitations.**
  - Data collection was done at the headquarter of the DCs where accessibility to WASH services and other COVID-19 interventions might be better than in remote settings.
  - Not every challenges reported apply to every kind of disability.
